# DNA barcoding of economically important freshwater fish species from north‐central Nigeria uncovers cryptic diversity

**DOI:** 10.1002/ece3.4210

**Published:** 2018-06-13

**Authors:** Oluyinka A. Iyiola, Lotanna M. Nneji, Moshood K. Mustapha, Chioma G. Nzeh, Segun O. Oladipo, Ifeanyi C. Nneji, Agboola O. Okeyoyin, Christopher D. Nwani, Obih A. Ugwumba, Adiaha A. A. Ugwumba, Emmanuel O. Faturoti, Yun‐yu Wang, Jing Chen, Wen‐Zhi Wang, Adeniyi C. Adeola

**Affiliations:** ^1^ Department of Zoology Faculty of Life Sciences University of Ilorin Ilorin Kwara State Nigeria; ^2^ Kunming Institute of Zoology Chinese Academy of Sciences Kunming China; ^3^ Sino‐Africa Joint Research Center Chinese Academy of Sciences Kunming China; ^4^ Kunming College of Life Science University of Chinese Academy of Sciences Kunming China; ^5^ Department of Biosciences and Biotechnology Kwara State University Malete Kwara State Nigeria; ^6^ Department of Biological Science University of Abuja Abuja Nigeria; ^7^ Nigerian National Park Service Headquarters Federal Capital Territory Abuja Nigeria; ^8^ Department of Zoology and Environmental Biology University of Nigeria Nsukka Nigeria; ^9^ Department of Zoology University of Ibadan Ibadan Oyo State Nigeria; ^10^ Department of Aquaculture and Fisheries Management University of Ibadan Ibadan Oyo State Nigeria; ^11^ Wild Forensic Center Kunming China

**Keywords:** Biodiversity, conservation policy, geographic variation, integrative taxonomy, mitochondrial DNA, population divergence

## Abstract

This study examines the utility of morphology and DNA barcoding in species identification of freshwater fishes from north‐central Nigeria. We compared molecular data (mitochondrial cytochrome *c* oxidase subunit I (*COI*) sequences) of 136 de novo samples from 53 morphologically identified species alongside others in GenBank and BOLD databases. Using DNA sequence similarity‐based (≥97% cutoff) identification technique, 50 (94.30%) and 24 (45.30%) species were identified to species level using GenBank and BOLD databases, respectively. Furthermore, we identified cases of taxonomic problems in 26 (49.00%) morphologically identified species. There were also four (7.10%) cases of mismatch in DNA barcoding in which our query sequence in GenBank and BOLD showed a sequence match with different species names. Using DNA barcode reference data, we also identified four unknown fish samples collected from fishermen to species level. Our Neighbor‐joining (NJ) tree analysis recovers several intraspecific species clusters with strong bootstrap support (≥95%). Analysis uncovers two well‐supported lineages within *Schilbe intermedius*. The Bayesian phylogenetic analyses of Nigerian *S. intermedius* with others from GenBank recover four lineages. Evidence of genetic structuring is consistent with geographic regions of sub‐Saharan Africa. Thus, cryptic lineage diversity may illustrate species’ adaptive responses to local environmental conditions. Finally, our study underscores the importance of incorporating morphology and DNA barcoding in species identification. Although developing a complete DNA barcode reference library for Nigerian ichthyofauna will facilitate species identification and diversity studies, taxonomic revisions of DNA sequences submitted in databases alongside voucher specimens are necessary for a reliable taxonomic and diversity inventory.

## 1 INTRODUCTION

Nigerian freshwater bodies include reservoirs, lakes, rivers, ponds, and perennial swamps which constitute about 12% of Nigeria’s total surface area (Ita, Sado, Balogun, Pandogori, & Ibitoye, 1985). They are richly endowed with fishery resources of more than 268 species of freshwater fishes (Froese & Pauly, 2017; Olaosebikan & Bankole, 2005; Olaosebikan & Raji, 1998). These serve enormous socio‐economic importance as sources of animal protein, income etc. However, over the years, reports have shown decline in the number of fish caught from most Nigerian inland waters (Oguntade, Oketoki, Ukenye, Usman, & Adeleke, 2014). This could be attributed to inadequate management of fisheries, climate change, pollution, and degradation of water bodies (Odo, Nwani, & Eyo, 2009). The impact of environmental pollution and other human activities on fish diversity cannot be overestimated. Hence, improved management plans and conservation approaches will aid in preventing loss of Nigerian fish diversity.

Accurate identification of species is a pivotal component in conservation efforts. The use of traditional methods (morphological characters) in species identification is common in Nigeria. In fact, about 48% of Nigerian freshwater fish species have been characterized using this method (Nwani et al., 2011). Although the use of morphological approach can be incorrect (Ward, Hanner, & Hebert, 2009), its accuracy has not yet been tested for Nigerian fishes. The challenges of the use of morphology lie in the discrimination of closely related organisms (Rasmussen, Morrissey, & Hebert, 2009). This has paved way for the development of improved molecular approaches for identification of fish species (Abdullah & Rehbein, 2017; Nazarov et al., 2012; Nwani, Eyo, & Udoh, 2016; Ratnasingham & Hebert, 2007).

Different DNA‐based methods, particularly DNA barcoding, have been evaluated for identification of fish species in Africa including Nigeria (Nwakanna, Ude, & Unachukwu, 2015; Nwani et al., 2011). DNA barcoding is a species identification tool, the information from which may be used as a conservation tool or potentially as part of the evidence to delimit species (Crawford, Alonso, Jaramillo, Sucre, & Ibáñez, 2011; Crawford et al., 2012; Gehring, Ratsoavina, & Vences, 2010; Nazarov et al., 2012; Vargas, Araújo, & Santos, 2009). It involves the use of 5′ region of the mitochondrial cytochrome *c* oxidase subunit I (*COI*) as a target gene (Hebert, Cywinska, Ball, & deWaard, 2003). DNA barcoding has been proven effective in facilitating rapid species identification when compared with morphological taxonomic approach (Hebert et al., 2003). For instances, in several studies, more than 98% of the analyzed species were clearly identified using DNA barcoding approach (Costa et al., 2012; deWaard, Hebert, & Humble, 2011; Liu & Zhang, 2018; Steinke, Zemlak, & Hebert, 2009; Valdez‐Moreno, Ivanova, Elías‐Gutiérrez, Contreras‐Balderas, & Hebert, 2009; Ward, Zemlak, Innes, Last, & Hebert, 2005; Zhang & Hanner, 2011). Studies have also documented the usefulness of DNA barcoding approach in unraveling cryptic lineages within many species of fish (Benzaquem, Oliveira, da Silva Batista, Zuanon, & Porto, 2015; Mat Jaafar, Taylor, Mohd Nor, de Bruyn, & Carvalho, 2012; Mohammed, Manal, Rasha, & Magdy, 2016). Thus, DNA barcoding could be an effective genetic tool that would assist Nigerian conservation managers in identifying species accurately and uncover hidden diversity.

To date, there are no studies on the DNA barcoding of freshwater fishes in north‐central region of Nigeria. Herein, we explored the use of DNA barcoding as reliable molecular tool for identification of fish species obtained from the north‐central Nigeria. We evaluated and compared GenBank and BOLD databases for use in species identification. Furthermore, we compared the taxonomic reliability of morphological method against DNA barcodes. Finally, we examined the usefulness of DNA barcode reference data in uncovering cryptic lineage diversity in fish species from north‐central Nigeria.

## 2 MATERIALS AND METHODS

### 2.1 Sample collection

We collected one hundred thirty‐six (136) freshwater fish samples belonging to 53 species between 2016 and 2017 (Table 1). Our sampling covered nine (9) inland water bodies (Oyun and Asa Reservoirs, Rivers Asa, Moro, Awon, Apodu‐Malete, Asa‐Laduba and Niger; and, Jebba Hydroelectric Power Dam Basin) in north‐central Nigeria. Specimens were caught using gill and cast nets. Fish specimens were identified from monographs, description checklist and standard taxonomic guides. Species identification and nomenclature followed fish identification guide of Olaosebikan and Raji (1998), Idodo‐Umeh (2003) and Fish Base databases (Froese & Pauly, 2017). Additional species identification and verification were sought from two trained taxonomists at the Department of Zoology, University of Ilorin, Nigeria. After each specimen was identified, tail fin and white muscle tissue were taken and preserved in 95% ethanol. The voucher specimens were fixed with 4% formalin and kept in 70% ethanol. Representative voucher specimens were imaged by digital scanning and later deposited in Museums of Department of Bioscience and Biotechnology, Kwara State University, Malete, Nigeria and the rest in the Department of Zoology, University of Ilorin, Nigeria. Furthermore, to verify the reliability of DNA barcode reference data in identification of unknown fish specimens, four muscle tissue samples of unknown fish species were collected from fishermen in River Niger, Niger State, Nigeria (Table 1). Morphological identification using fish guide could not be achieved due to unavailability of whole fish specimens. We preserved tissues in 95% ethanol and subsequently stored under −80°C.

**Table 1:** List of species including voucher’s specimen number, species name, locality information and GenBank accession number

S/No	Specimen voucher	Organism	Locality	GenBank accession no.
1	YLMC112	*Alestes baremoze*	Nigeria: Niger State, Jebba HEP Upstream	MG824552
2	YLMC145	*A. baremoze*	Nigeria: Kwara State, Jebba HEP Downstream	MG824553
3	YLMC165	*Auchenoglanis biscutatus*	Nigeria: Niger State, Jebba HEP Upstream	MG824554
4	YLMC153	*Auchenoglanis occidentalis*	Nigeria: Niger State, Jebba HEP Upstream	MG824555
5	YLMC025	*A. occidentalis*	Nigeria: Kwara State, Moro River	MG824556
6	YLMC042	*A. occidentalis*	Nigeria: Kwara State, Moro River	MG824557
7	YLMC281	*A. occidentalis*	Nigeria: Kwara State, Moro River	–
8	YLMC274	*Bagrus bajad*	Nigeria: Kwara State, Jebba HEP Downstream	MG824559
9	YLMC061	*B. bajad*	Nigeria: Kwara State, Jebba HEP Downstream	MG824560
10	YLMC210	*Bagrus docmac*	Nigeria: Niger State, Jebba HEP Upstream	MG824561
11	YLMC051	*B. docmac*	Nigeria: Niger State, Jebba HEP Upstream	MG824562
12	YLMC149	*Brienomyrus niger*	Nigeria: Niger State, Jebba HEP Upstream	MG824563
13	YLMC022	*B. niger*	Nigeria: Kwara State, Moro River	MG824564
14	YLMC233	*Brycinus nurse*	Nigeria: Kwara State, Jebba HEP Downstream	MG824565
15	YLMC097	*B. nurse*	Nigeria: Kwara State, Jebba HEP Downstream	MG824566
16	YLMC312	*Brycinus* sp.	Nigeria: Niger State, Jebba HEP Upstream	MG824567
17	YLMC213	*Chrysichthys cf auratus*	Nigeria: Niger State, Jebba HEP Upstream	MG824568
18	YLMC062	*C. cf auratus*	Nigeria: Niger State, Jebba HEP Upstream	MG824569
19	YLMC295	*Chrysichthys nigrodigitatus*	Nigeria: Niger State, Jebba HEP Upstream	MG824570
20	YLMC315	*C. nigrodigitatus*	Nigeria: Niger State, Jebba HEP Upstream	MG824571
21	YLMC002	*Chrysichthys* sp.	Nigeria: Kwara State, Oyun Reservoir	MG824572
22	YLMC006	*Chrysichthys* sp.	Nigeria: Kwara State, Oyun Reservoir	MG824573
23	YLMC461	*Chrysichthys* sp.	Nigeria: Niger State, Jebba HEP Upstream	MG824574
24	YLMC081	*C. citharus*	Nigeria: Niger State, Jebba HEP Upstream	MG824575
25	YLMC076	*C. citharus*	Nigeria: Niger State, Jebba HEP Upstream	MG824576
26	YLMC079	*C. citharus*	Nigeria: Niger State, Jebba HEP Upstream	MG824577
27	YLMC092	*C. citharus*	Nigeria: Niger State, Jebba HEP Upstream	MG824578
28	YLMC463	*Clarias gabonensis*	Nigeria: Niger State, Jebba HEP Upstream	MG824579
29	YLMC209	*C. gariepinus*	Nigeria: Niger State, Jebba HEP Upstream	MG824580
30	YLMC240	*C. gariepinus*	Nigeria: Kwara State, Apodu‐Malete	MG824581
31	YLMC273	*C. gariepinus*	Nigeria: Kwara State, Apodu‐Malete	MG824582
32	YLMC084	*C. gariepinus*	Nigeria: Niger State, Jebba HEP Upstream	MG824583
33	YLMC115	*C. laticeps*	Nigeria: Niger State, Jebba HEP Upstream	MG824584
34	YLMC154	*C. laticeps*	Nigeria: Niger State, Jebba HEP Upstream	MG824585
35	YLMC197	*C. laticeps*	Nigeria: Niger State, Jebba HEP Upstream	MG824586
36	YLMC300	*Distichodius engycephalus*	Nigeria: Kwara State, Jebba HEP Downstream	MG824587
37	YLMC134	*D. rostratus*	Nigeria: Niger State, Jebba HEP Upstream	MG824588
38	YLMC301	*D. rostratus*	Nigeria: Niger State, Jebba HEP Upstream	MG824589
39	YLMC098	*D. rostratus*	Nigeria: Niger State, Jebba HEP Upstream	MG824590
40	YLMC318	*Gymnarchus niloticus*	Nigeria: Niger State, Jebba HEP Upstream	MG824591
41	YLMC333	*G. niloticus*	Nigeria: Niger State, Jebba HEP Upstream	MG824592
42	YLMC013	*Hemichromis bimaculatus*	Nigeria: Kwara State, Asa River	MG824593
43	YLMC021	*H. bimaculatus*	Nigeria: Kwara State, Asa River	MG824594
44	YLMC010	*Heterotis niloticus*	Nigeria: Kwara State, Asa River	MG824595
45	YLMC284	*H. niloticus*	Nigeria: Kwara State, Asa River	–
46	YLMC180	*Hydrocynus vittatus*	Nigeria: Niger State, Jebba HEP Upstream	MG824597
47	YLMC083	*H. vittatus*	Nigeria: Niger State, Jebba HEP Upstream	MG824598
48	YLMC222	*Hyperopisus bebe*	Nigeria: Niger State, Jebba HEP Upstream	MG824599
49	YLMC033	*H. bebe*	Nigeria: Kwara State, Awon River	MG824600
50	YLMC090	*Labeo coubie*	Nigeria: Kwara State, Jebba HEP Downstream	MG824601
51	YLMC121	*L.coubie*	Nigeria: Niger State, Jebba HEP Upstream	MG824602
52	YLMC127	*L.coubie*	Nigeria: Niger State, Jebba HEP Upstream	MG824603
53	YLMC031	*L. parvus*	Nigeria: Kwara State, Awon River	MG824604
54	YLMC032	*L. parvus*	Nigeria: Kwara State, Awon River	MG824605
55	YLMC100	*L. senegalensis*	Nigeria: Kwara State, Jebba HEP Downstream	MG824606
56	YLMC035	*L. senegalensis*	Nigeria: Niger State, River Niger	MG824607
57	YLMC054	*L. senegalensis*	Nigeria: Kwara State, Jebba HEP Downstream	MG824608
58	YLMC138	*L. niloticus*	Nigeria: Niger State, Jebba HEP Upstream	MG824609
59	YLMC193	*L. niloticus*	Nigeria: Niger State, Jebba HEP Upstream	MG824610
60	YLMC082	*L. niloticus*	Nigeria: Niger State, Jebba HEP Upstream	MG824611
61	YLMC244	*Malapterurus* sp.	Nigeria: Kwara State, Jebba HEP Downstream	MG824612
62	YLMC225	*Marcusenius senegalensis*	Nigeria: Kwara State, Jebba HEP Downstream	MG824613
63	YLMC335	*M. senegalensis*	Nigeria: Kwara State, Jebba HEP Downstream	MG824614
64	YLMC454	*M. senegalensis*	Nigeria: Kwara State, Jebba HEP Downstream	MG824615
65	YLMC455	*M. senegalensis*	Nigeria: Kwara State, Jebba HEP Downstream	MG824616
66	YLMC053	*M. senegalensis*	Nigeria: Kwara State, Jebba HEP Downstream	MG824617
67	YLMC036	*Mormyrops anguilloides*	Nigeria: Niger State, River Niger	MG824618
68	YLMC024	*M. anguilloides*	Nigeria: Kwara State, Moro River	MG824619
69	YLMC269	*M. anguilloides*	Nigeria: Niger State, Jebba HEP Upstream	MG824620
70	YLMC289	*M. anguilloides*	Nigeria: Niger State, Jebba HEP Upstream	–
71	YLMC046	*Mormyrus hasselquistii*	Nigeria: Niger State, River Niger	MG824622
72	YLMC381	*Mormyrus macrophthalmus*	Nigeria: Kwara State, Jebba HEP Downstream	MG824623
73	YLMC091	*M. macrophthalmus*	Nigeria: Kwara State, Jebba HEP Downstream	MG824624
74	YLMC039	*Mormyrus tapirus*	Nigeria: Niger State, River Niger	MG824625
75	YLMC217	*M. tapirus*	Nigeria: Kwara State, Jebba HEP Downstream	MG824626
76	YLMC172	*Oreochromis aureus*	Nigeria: Kwara State, Jebba HEP Downstream	MG824627
77	YLMC206	*O. aureus*	Nigeria: Kwara State, Jebba HEP Downstream	MG824628
78	YLMC218	*O. aureus*	Nigeria: Kwara State, Jebba HEP Downstream	MG824629
79	YLMC008	*Oreochromis* sp.	Nigeria: Kwara State, Asa River	MG824630
80	YLMC126	*Oreochromis* sp.	Nigeria: Kwara State, Jebba HEP Downstream	MG824631
81	YLMC096	*Oreochromis* sp.	Nigeria: Kwara State, Jebba HEP Downstream	MG824632
82	YLMC004	*Parachanna insignis*	Nigeria: Kwara State, Oyun Reservoir	MG824633
83	YLMC453	*P. insignis*	Nigeria: Kwara State, Kwara State, Asa‐Laduba	MG824634
84	YLMC005	*Parachanna obscura*	Nigeria: Kwara State, Oyun Reservoir	MG824635
85	YLMC045	*P. obscura*	Nigeria: Kwara State, Kwara State, Asa‐Laduba	MG824636
86	YLMC317	*Protopterus* sp.	Nigeria: Kwara State, Jebba HEP Downstream	MG824637
87	YLMC001	*Sarotherodon galilaeus*	Nigeria: Kwara State, Oyun Reservoir	MG824638
88	YLMC446	*S. galilaeus*	Nigeria: Kwara State, Apodu‐Malete	MG824639
89	YLMC007	*Schilbe intermedius*	Nigeria: Kwara State, Oyun Reservoir	MG824640
90	YLMC009*	*S. intermedius*	Nigeria: Kwara State, Asa reservoir	MG824641
91	YLMC016*	*S. intermedius*	Nigeria: Kwara State, Asa reservoir	MG824642
92	YLMC017*	*S. intermedius*	Nigeria: Kwara State, Asa reservoir	MG824643
93	YLMC271*	*S. intermedius*	Nigeria: Kwara State, Jebba HEP Downstream	MG824644
94	YLMC285*	*S. intermedius*	Nigeria: Niger State, Jebba HEP Upstream	MG824645
95	YLMC034*	*S. intermedius*	Nigeria: Kwara State, Kwara State, Awon River	MG824646
96	YLMC450*	*S. intermedius*	Nigeria: Kwara State, Kwara State, Asa‐Laduba	MG824647
97	YLMC451*	*S. intermedius*	Nigeria: Kwara State, Kwara State, Asa‐Laduba	MG824648
98	YLMC277*	*S. intermedius*	Nigeria: Kwara State, Kwara State, Asa‐Laduba	–
99	YLMC139*	*Schilbe mystus*	Nigeria: Kwara State, Jebba HEP Downstream	MG824650
100	YLMC216*	*S. mystus*	Nigeria: Kwara State, Jebba HEP Downstream	MG824651
101	YLMC248*	*Schilbe* sp.	Nigeria: Kwara State, Jebba HEP Downstream	MG824652
102	YLMC123	*Synodontis* aff. *bastiani*	Nigeria: Kwara State, Jebba HEP Downstream	MG824653
103	YLMC063	*S. *aff. *bastiani*	Nigeria: Kwara State, Jebba HEP Downstream	MG824654
104	YLMC152	*S. *aff. *bastiani*	Nigeria: Kwara State, Jebba HEP Downstream	MG824655
105	YLMC299	*S. *aff. *bastiani*	Nigeria: Kwara State, Jebba HEP Downstream	MG824656
106	YLMC099	*S. *aff. *bastiani*	Nigeria: Kwara State, Jebba HEP Downstream	MG824657
107	YLMC029	*Synodontis* aff. *haugi*	Nigeria: Kwara State, Awon River	MG824658
108	YLMC030	*S. *aff. *haugi*	Nigeria: Kwara State, Awon River	MG824659
109	YLMC125	*Synodontis batesonda*	Nigeria: Kwara State, Jebba HEP Downstream	MG824660
110	YLMC103	*Synodontis clarias*	Nigeria: Kwara State, Jebba HEP Downstream	MG824661
111	YLMC293	*S. clarias*	Nigeria: Kwara State, Jebba HEP Downstream	MG824662
112	YLMC040	*S. clarias*	Nigeria: Niger State, River Niger	MG824663
113	YLMC041	*S. clarias*	Nigeria: Niger State, River Niger	MG824664
114	YLMC106	*Synodontis membranacea*	Nigeria: Kwara State, Jebba HEP Downstream	MG824665
115	YLMC247	*S. membranacea*	Nigeria: Kwara State, Jebba HEP Downstream	MG824666
116	YLMC064	*S. membranacea*	Nigeria: Kwara State, Jebba HEP Downstream	MG824667
117	YLMC184	*Synodontis nigrita*	Nigeria: Niger State, Jebba HEP Upstream	MG824668
118	YLMC292	*S. nigrita*	Nigeria: Niger State, Jebba HEP Upstream	MG824669
119	YLMC108	*Synodontis obesus*	Nigeria: Kwara State, Jebba HEP Downstream	MG824670
120	YLMC069	*S. obesus*	Nigeria: Kwara State, Jebba HEP Downstream	MG824671
121	YLMC306	*Synodontis ocellifer*	Nigeria: Kwara State, Jebba HEP Downstream	MG824672
122	YLMC205	*Synodontis* sp.	Nigeria: Kwara State, Jebba HEP Downstream	MG824673
123	YLMC014	*Synodontis violacea*	Nigeria: Kwara State, Asa river	MG824674
124	YLMC027	*S. violacea*	Nigeria: Kwara State, Asa river	MG824675
125	YLMC028	*S. violacea*	Nigeria: Kwara State, Asa river	MG824676
126	YLMC437	*S. violacea*	Nigeria: Niger State, Jebba HEP Upstream	MG824677
127	YLMC057	*S. violacea*	Nigeria: Niger State, Jebba HEP Upstream	MG824678
128	YLMC161	*Tetraodon lineatus*	Nigeria: Niger State, Jebba HEP Upstream	MG824679
129	YLMC268	*T. lineatus*	Nigeria: Kwara State, Jebba HEP Downstream	MG824680
130	YLMC070	*Tetraodon lineatus*	Nigeria: Niger State, Jebba HEP Upstream	MG824681
131	YLMC011	*Tilapia zillii*	Nigeria: Kwara State, Asa reservoir	MG824682
132	YLMC020	*T. zillii*	Nigeria: Kwara State, Asa reservoir	MG824683
133	YLMC263	*T. zillii*	Nigeria: Kwara State, Jebba HEP Downstream	MG824684
134	YLMC068	*T. zillii*	Nigeria: Kwara State, Jebba HEP Downstream	MG824685
135	YLMC272	*T. zillii*	Nigeria: Kwara State, Jebba HEP Downstream	–
136	YLMC354	*T. zillii*	Nigeria: Kwara State, Jebba HEP Downstream	–
Sample 1	YLMC050^a^	*A. occidentalis*	Collected from a fisherman beside River Niger, Nigeria	MG824558
Sample 2	YLMC047^a^	*H. niloticus*	Collected from a fisherman beside River Niger, Nigeria	MG824596
Sample 3	YLMC048^a^	*M. anguilloides*	Collected from a fisherman beside River Niger, Nigeria	MG824621
Sample 4	YLMC049^a^	*S. intermedius*	Collected from a fisherman beside River Niger, Nigeria	MG824649

*Notes*

HEP: hydroelectric plant; −: samples for which PCR amplification failed.

Vouchers with asterisks represent samples deposited in the Museums of Department of Bioscience and Biotechnology, Kwara State University, Malete, Nigeria.

a Unknown tissue samples from collected from a fisherman River Niger, Nigeria.

### 2.2 DNA extraction, polymerase chain reaction (PCR), amplification and sequencing

We used proteinase K to digest the ethanol‐preserved tissues and followed the standard phenol‐chloroform extraction procedure to extract the total genomic DNA (Sambrook & Russell, 2001). The concentration of the extracted DNA estimated using a UV spectrophotometer ranged from 91.2 to 6905.8 ng/μl. For the preparation of genomic working DNA, we diluted the DNA extracts with sterile water to obtain genomic working DNA with concentration ranging from 30.0 to 80.0 ng/μl. After, we amplified the mitochondrial DNA Cytochrome *c* Oxidase I (*COI*) gene of the newly acquired specimens in a volume reaction of 25 μl that contained 1.5 μl of genomic working DNA, 18.5 μl of PCR water, 2.5 μl of Taq polymerase buffer, 2 μl of dNTP, 1 μl of each of the forward and reverse primers (10 pm/μl) and 0.30 μl of rTaq polymerase. The primers used for the amplification were designed by Ward et al. (2005): FishF1 – 5′TCAACCAACCACAAAGACATTGGCAC3′ and FishR1‐5′TAGACTTCTGGGTGGCCAAAGAATCA3′. The PCR cycle profiles were as follow: 5 min initial denaturation at 94°C, followed by 35 cycles of 1 min at 94°C, annealing for 45 s at 55°C, extension for 1 min at 72°C; final extension for 10 min at 72°C. Purified PCR products were directly sequenced in both forward and reverse directions with an automated DNA sequencer (ABI 3730) following manufacturer’s instruction.

### 2.3 Sequence assembly and data analyses

The nucleotide sequences were viewed and confirmed by eye using SeqManTMII (DNASTAR Lasergene 7). They were aligned in MEGA 7.0 using ClustalW (Kumar, Stecher, & Tamura, 2016) with default parameters. The aligned sequences were translated into amino acids to check for premature stop codons and to confirm that the open reading frame was maintained in the protein‐coding loci. To confirm the identity of the amplified sequences, we conducted BLAST searches by inputting the FASTA sequences in the nucleotide collection database (under option “other”) for each specimen using the Megablast search for highly similar sequences on GenBank (https://blast.ncbi.nlm.nih.gov/Blast.cgi). Additionally, FASTA sequences of each of *COI* sequences were inputted into the BOLD Identification Request tool (http://www.boldsystems.org/index.php/IDS_OpenIdEngine). Sequences were submitted for species level identification under option “Species Level Barcode Record.” Following Hebert et al. (2003), a similarity cutoff of ≥97% was used for species level identification for sequences submitted to both GenBank and BOLD databases. The submitted sequence was matched to a species with the highest similarity score. We further compared species names assigned using morphology, GenBank and BOLD databases. Using BOLD database, we estimated the Barcode Index Number (BIN), average and maximum intraspecific distance, average genetic distance to the nearest neighbor and the nearest neighbor member for each species.

We used MEGA v. 7.0 to create a neighbor‐joining (NJ) tree based on the Kimura 2 parameter distance (K2P) (Kimura, 1980) and estimated the intergeneric, inter‐ and intraspecific sequence divergences. For the NJ tree, we considered bootstrap values of 95% and above as strongly supported. Following Decru, Van Ginneken, Verheyen, and Snoeks (2016), identification is considered successful if the sequence and the match are conspecific and failed if they are allospecific.

Upon discovery of deeply divergent lineages within species, further genetic analysis was carried out to investigate possibility of cryptic lineage diversity. To infer this, we downloaded additional related sequences of such species from the GenBank (Table S1). The Bayesian Inference (BI) analysis was rooted with a closely related species as out‐group taxon. We partitioned the *COI* gene into codon position 1, 2 and 3. Evolutionary model testing for each of the partitioned codon was performed using JMODELTEST (Posada, 2008). Furthermore, models were selected: GTR + G for the first and third codon positions; and F81 for the second codon position. Phylogenetic relationships were evaluated using a Bayesian framework as implemented in BEAST v1.6.1 (Drummond & Rambaut, 2007). Analysis was run for 20 million generations with sampling every 1,000th generation. Two independent runs with four Markov chain Monte Carlo Chains (MCMC) were performed. We excluded the first 25% of the tree as burn‐in before the log‐likelihood scores stabilized. A 50% majority rule consensus of the sampled trees was constructed and visualized using FigTree v1.4.2 (Rambaut, 2012). We considered bootstrap values of Bayesian Posterior Probabilities (PP) ≥0.95 as being strongly supported (Hillis & Huelsenbeck, 1992).

## 3 RESULTS

### 3.1 Morphology‐based species identification

Of the 136 specimens collected, all specimens (100%) were identified to consist of 53 species belonging to 28 genera and 18 families based on morphology (Table 2). This included 46 (86.80%) species identified to species level and seven (13.20%) species that could not be assigned species level and thus referred to genus.

**Table 2:** Species identification using morphological and DNA barcode approaches. Species identification cutoff of 97% was used for GenBank and BOLD databases

S/No.	Morphological ID	GenBank identificationSpecies name (accession no)	Similarity (%)	BOLD identificationSpecies name	Similarity (%)
1	*Alestes baremoze*	*A. baremoze* (JF800979)	97	*A. baremoze*	100
2	*Auchenoglanis biscutatus*	*Auchenoglanis biscutatus* (JF510501)	100	*Auchenoglanis biscutatus*	100
3	*Auchenoglanis occidentalis*	*A. occidentalis* (HM882801)	100	*A. occidentalis*	100
4	*Bagrus bajad*	*B. bajad* (HM882795)	100	*B. bajad*	100
5	*Bagrus docmac*	*Bagrus docmac* (EU490857)	99	*Bagrus docmac*	99.20
6	*Hyperopisus bebe*	*Brienomyrus niger* (JF510502)	99	*B. niger* 1	99.80
7	*Brycinus nurse*	*Brycinus nurse* (HM882786)	99	*Brycinus nurse*	100
8	*Brycinus* sp.	*Brycinus* sp. (JF510504)	99	*Brycinus* sp.	99.26
9	*Chrysichthys* cf. *auratus*	*Chrysichthys* cf. *auratus* (HG803482)	100	N/A	N/A
10	*Chrysichthys nigrodigitatus*	*Chrysichthys nigrodigitatus* (HG803416)	100	*Chrysichthys auratus*	100
11	*Chrysichthys* sp.	*Chrysichthys* sp. (HG803490)	99	*Chrysichthys* sp.^2^	99.60
12	*Citharinus citharus*	*Citharinus citharus citharus* (HM882705)	100	*Citharinus citharus* 3	100
13	*Clarias gabonensis*	*Clarias gabonensis* (HM882836)	100	*Clarias gabonensis* 4	100
14	*Clarias gariepinus*	*Clarias gariepinus* (HM882821)	100	*Clarias gariepinus* 5	100
15	*Clarotes laticeps*	*Clarotes laticeps* (HG803491)	100	*Clarotes laticeps*	100
16	*Distichodius engycephalus*	*Distichodius engycephalus* (HM882993)	99	*Distichodius engycephalus*	99.80
17	*Distichodius rostratus*	*Distichodius rostratus* (HM882994)	100	*Distichodius rostratus*	100
18	*Gymnarchus niloticus*	*G. niloticus* (AP009610)	100	*G. niloticus*	100
19	*Hemichromis bimaculatus*	*Hemichromis bimaculatus* (HM882913)	99	*Hemichromis bimaculatus* 6	99.80
20	*Hydrocynus vittatus*	*Hydrocynus vittatus* (HM882886)	100	*Hydrocynus vittatus*	100
21	*Heterotis niloticus*	*H. niloticus* (FJ890318)	100	*H. niloticus*	100
22	*H. bebe*	*H. bebe* (JF510502)	99	*B. niger* 7	99.80
23	*Labeo coubie*	*Labeo* sp. (HM882842)	100	*Labeo* sp.^8^	100
24	*Labeo parvus*	*Labeo parvus* (AP013339)	100	*Labeo parvus* 9	100
25	*Labeo senegalensis*	*Labeo horie* (JX074211)	100	*Labeo horie* 10	100
26	*Lates niloticus*	*L. niloticus* (KJ443710)	99	*L. niloticus*	99.80
27	*Malapterurus* sp.	*Malapterurus melanochir* (KT193322)	93	N/A	N/A
28	*Marcusenius senegalensis*	*Marcusenius senegalensis* (HM882721)	99	*Marcusenius senegalensis*	99.80
29	*Mormyrops anguilloides*	*M. anguilloides* (AP011576)	99	*M. anguilloides*	99.60
30	*Mormyrus rume*	*Mormyrus hasselquistii* (HM882746)	100	*Mormyrus hasselquistii* 11	100
31	*Mormyrus macrophthalmus*	*Mormyrus macrophthalmus* (HM882759)	100	*Mormyrus macrophthalmus*	100
32	*Mormyrus rume*	*Mormyrus tapirus* (HM882745)	99	*Mormyrus longirostris* 12	100
33	*Oreochromis aureus*	*Sarotherodon galilaeus* (HM882887)	100	*Sarotherodon galilaeus* 13	100
34	*Oreochromis* sp.	*Oreochromis* sp. (KX781822)	100	*Oreochromis aureus* 14	100
35	*Parachanna insignis*	*Parachanna obscura* (MF496976)	100	*Parachanna obscura* 15	100
36	*Parachanna obscura*	*Parachanna obscura* (MF496976)	100	*Parachanna obscura* 16	100
37	*Protopterus* sp.	*Protopterus* sp. (JF510519)	99	*Protopterus* sp.	99.10
38	*Oreochromis niloticus*	*Sarotherodon galilaeus* (KM438546)	99	*Sarotherodon galilaeus* 17	100
39	*Schilbe intermedius*	*S. intermedius* (HM882935)	100	*S. intermedius*	100
40	*Schilbe* sp.	*S. intermedius* (KT193441)	95	*S. intermedius*	98.90
41	*Synodontis eupterus*	*Synodontis* aff. *bastiani* (HF565861)	99	*Synodontis* aff. *bastiani* 18	100
42	*Synodontis schall*	*Synodontis* aff*. haugi* (HF565896)	100	*Synodontis* aff*. haugi* 19	100
43	*Synodontis batensoda*	*Synodontis batensoda* (HF565863)	100	*Synodontis batensoda* 20	100
44	*Synodontis clarias*	*Synodontis clarias* (HF565870)	99	*Synodontis clarias*	99.80
45	*Synodontis membranacea*	*Synodontis membranacea* (HF565908)	100	*Synodontis membranacea* 21	100
46	*Schilbe mystus*	*Schilbe mystus* (HM882942)	99	*Schilbe mystus*	100
47	*Synodontis nigrita*	*Synodontis nigrita* (HF565916)	100	*Synodontis nigrita*	100
48	*Synodontis obesus*	*Synodontis obesus* (HF565926)	99	*Synodontis obesus* 22	99.30
49	*Synodontis ocellifer*	*Synodontis* sp. (HM882967)	100	*Synodontis* sp.^23^	100
50	*Synodontis* sp.	*Synodontis batensoda* (HF565863)	96	N/A	N/A
51	*Synodontis violacea*	*Synodontis violacea* (HF565985)	99	*Synodontis* sp.^24^	100
52	*Tetraodon lineatus*	*Tetraodon lineatus* (KT715694)	100	*Tetraodon lineatus* 25	100
53	*Tilapia guineensis*	*Coptodon zillii* (KJ938220)	100	*Tilapia zillii* 26	100

*Note*

N/A, Sequences for the specimen are not available in BOLD; Individuals with superscript before species name represent specimens for which a species level match could not be made, but the queried species is likely to be one of the following: ^1^ *B. niger* or *H. bebe*; ^2^ *Chrysichthys* sp. or *C. nigrodigitatus*; ^3^ *Citharinus citharus* or *C. citharus citharus*; ^4^ *Clarias gabonensis*,* Clarias* sp. or *C. agboyiensis*; ^*5*^ *C. gariepinus* or *Clarias* sp.; ^6^ *Hemichromis bimaculatus* or *H. fasciatus*; ^7^ *B. niger* or *H. bebe*; ^8^ *Labeo* sp. or *L. coubie*; ^9^ *L. parvus*,* L. *cf*. parvus*,* L. cylindricus*,* L. victorianus*,* Labeobarbus altianalis* or *L. molybdinus*; ^10^ *L. horie* or *L. senegalensis*; ^11^ *Mormyrus hasselquistii* or *Mormyrus* sp.; ^12^ *M. longirostris*,* M. tapirus* or *M. rume*; ^13^ *Sarotherodon galilaeus*,* Sarotherodon* sp., *Oreochromis mossambicus*,* O. niloticus*,* Oreochromis* sp., *O. aureus*,* O. aureus *×* O. niloticus*,* Oreochromis* sp. TP or *Tilapia zillii*; ^14^ *Oreochromis mossambicus*,* O. niloticus*,* Oreochromis* sp., *O. aureus*,* O. aureus *×* O. niloticus*,* Oreochromis* sp. TP or *Tilapia zillii*; ^15^ *Parachanna obscura* or *P. insignis*; ^16^ *P. obscura* or *P. insignis*; ^17^ *S. galilaeus*,* O. mossambicus*,* Sarotherodon* sp., *O. leucostictus*,* O. niloticus*,* Oreochromis* sp., *O. aureus*,* O. aureus *×* O. niloticus*,* Oreochromis* sp. TP or *S. galilaeus boulengeri*; ^18^ *Synodontis* aff*. bastiani*,* S. schall*,* S. *aff*. schall*,* S. *aff*. haugi* or *S. ouemeensis*; ^19^ *S. *aff*. haugi*,* S. *aff*. schall*,* S. *aff. *bastiani*,* S. schall* or *S. ouemeensis*; ^20^ *S. batensoda*,* S. resupinatus*,* S. membranacea*,* Brachysynodontis batensoda* or *S. *aff. *schall*; ^21^ *S. membranacea*,* B. batensoda*,* S. *aff. *schall*,* S. batensoda* or *S. resupinatus*; ^22^ *S. obesus*,* S. *cf*. obesus* or *S. rebeli*; ^23^ *Synodontis* sp. or *S. ocellifer*; ^24^ *Synodontis* sp. or *S. violaceus*; ^25^ *Tetraodon lineatus* or *T. pustulatus*; ^26^ *T. zillii*,* O. mossambicus*,* Coptodon zillii*,* Coptodon* sp., *C. rendalli* or *T. guineensis*.

### 3.2 Amplification success and sequence statistics

We obtained 130 sequences (all >500 bp) belonging to 53 morphologically identified species (Table 1). This accounts for 95.60% amplification success rate (Table 1). Even with repeated attempts, sequences of six samples did not amplify probably due to some technical problems. However, we did not perform multiple temperature gradients or used alternative primers for PCR amplification for failed samples, as this would increase laboratory cost, time and resources. After trimming ambiguous bases, overall consensus length of 537 nucleotide base pairs (bp) was used in the analyses. The sequences contained 287 conserved sites, 260 variable sites and 250 phylogenetically informative sites. Overall base contents were as follows: A = 25.90%, C = 28.90%, G = 17.00% and T = 28.20%. No insertion, deletions and stop codon were observed; hence, all the amplified sequences represent functional mitochondrial *COI* sequences. Novel sequences generated were deposited in GenBank (Table 1) under Accession Nos. MG824552–MG824685. The *COI* sequences and related information for each specimen were also made publicly accessible via the BOLD systems website within “Diversity studies and DNA barcoding of Nigerian freshwater and marine fishes” project as part of the international fish barcode of life project.

### 3.3 DNA sequence similarity‐based species identification

All the 130 successfully amplified sequences were crossreferenced to GenBank and BOLD databases. One hundred twenty‐seven sequences (97.70%) belonging to 50 (94.30%) species showed species sequence similarity of ≥97% when crossreferenced in the GenBank. However, individuals morphologically identified as *Malapterurus* sp., *Schilbe* sp. and *Synodontis* sp. could not be identified to species level (DNA similarity sequences of ≤97%) using GenBank database. Using BOLD, 63 sequences (48.50%) belonging to 24 species (45.30%) could be matched to species level. Result shows that nearest neighbor values of the sequences were higher than the maximum intraspecific distance, pointing to the presence of a barcoding gap (Table 3). On the other hand, species level match for remaining 63 (48.50%) sequences obtained from 26 morphologically identified species (49.00%) could not be made, as queried specimens showed sequence similarity of ≥97% for more than one species. Attempt to estimate the BIN for these sequences failed, indicating possible taxonomic problems (Table 3). Furthermore, four sequences (3.00%) of three species (5.70%) morphologically identified as *Chrysichthys* cf. *auratus*,* Malapterurus* sp. and *Synodontis* sp. were unable to match to any records in BOLD database.

**Table 3:** Barcode Index Number details of freshwater fishes from north‐central Nigeria

Morphological ID	BOLD identificationSpecies name	*N*	BIN details				Nearest neighbor
			BIN	*AvD*	*MxD*	*DNN*	
Alestidae							
*Alestes baremoze*	*A. baremoze*	2	BOLD: AAJ3269	0.23	0.46	4.18	*Alestes dentex*
*Brycinus nurse*	*Brycinus nurse*	2	BOLD: AAI8453	0.47	1.08	11.4	*Brachyalestes bimaculatus*
*Brycinus* sp.	*Brycinus* sp.	1	BOLD: AAL6091			4.72	*Brycinus* cf. *macrolepidotus*
*Hydrocynus vittatus*	*Hydrocynus vittatus*	2	BOLD: AAE1861	0.61	0.16	5.14	*Hydrocynus forskahlii*
Arapaimidae							
*Heterotis niloticus*	*H. niloticus*	1	BOLD: AAL6307	0.00	0.00	16.85	*Monopterus albus*
Bagridae							
*Bagrus bajad*	*B. bajad*	2	BOLD: AAL7132	0.31	0.31	2.57	*Bagrus filamentosus*
*Bagrus docmac*	*Bagrus docmac*	2	BOLD: AAE1929	0.00	0.00	3.64	*B. bajad*
Channidae							
*Parachanna insignis*	*Parachanna obscura*	2	C/A				
*Parachanna obscura*	*Parachanna obscura*	2	C/A				
Cichlidae							
*Hemichromis bimaculatus*	*Hemichromis bimaculatus*	2	C/A				
*Oreochromis aureus*	*Sarotherodon galilaeus*	3	C/A				
*Oreochromis* sp.	*Oreochromis* sp.	3	C/A				
*Oreochromis niloticus*	*Sarotherodon galilaeus*	2	C/A				
*Tilapia guineensis*	*Tilapia zillii*	4	C/A				
Citharinidae							
*Citharinus citharus*	*Citharinus citharus*	4	C/A				
Clariidae							
*Clarias gabonensis*	*Clarias gabonensis*	1	C/A				
*Clarias gariepinus*	*Clarias gariepinus*	4	C/A				
Claroteidae							
*Auchenoglanis biscutatus*	*Auchenoglanis biscutatus*	1	BOLD: AAJ9618	0.00	0.00	8.03	*Auchenoglanis occidentalis*
*A.occidentalis*	*A. occidentalis*	3	BOLD: AAL5844	0.09	0.15	5.78	*A. occidentalis*
*Chrysichthys* cf. *auratus*	N/A	2	N/A				
*Chrysichthys nigrodigitatus*	*Chrysichthys auratus*	2	BOLD: AAL6568	0.08	0.16	3.37	*Chrysichthys nigrodigitatus*
*Chrysichthys* sp.	*Chrysichthys* sp.	3	C/A				
*Clarotes laticeps*	*Clarotes laticeps*	3	BOLD: AAK5763	0.28	0.80	13.80	*Chrysichthys nigrodigitatus*
Cyprinidae							
*Labeo coubie*	*Labeo* sp.	3	C/A				
*Labeo parvus*	*Labeo parvus*	2	C/A				
*Labeo senegalensis*	*Labeo horie*	3	C/A				
Distochodontidae							
*Distichodius engycephalus*	*Distichodius engycephalus*	1	BOLD: AAL6656			4.65%	*Distichodus petersii*
*Distichodius rostratus*	*Distichodius rostratus*	3	BOLD: AAL6019	0.23	0.61	5.14	*Distichodus engycephalus*
Gymnarchidae							
*Gymnarchus niloticus*	*G. niloticus*	2	BOLD: AAI6415	0.10	0.15	15.73	*Mastomys*
Latidae							
*Lates niloticus*	*L. niloticus*	3	BOLD: AAA2960	0.27	0.68	4.49	
Malapteruridae							
*Malapterurus* sp.	N/A	1					
Mochokidae							
*Synodontis eupterus*	*Synodontis* aff*. bastiani*	5	C/A				
*Synodontis schall*	*Synodontis* aff*. haugi*	2	C/A				
*Synodontis batensoda*	*Synodontis batensoda*	1	C/A				
*Synodontis clarias*	*Synodontis clarias*	4	BOLD: ACH9809	0.32	0.65	4.43	*Synodontis sorex*
*Synodontis membranacea*	*Synodontis membranacea*	3	C/A				
*Synodontis nigrita*	*Synodontis nigrita*	2	BOLD: AAL5812	0.29	1.08	3.53	*Synodontis*
*Synodontis obesus*	*Synodontis obesus*	2	C/A				
*Synodontis ocellifer*	*Synodontis* sp.	1	C/A				
*Synodontis* sp.	N/A	1					
*Synodontis violacea*	*Synodontis* sp.	5	C/A				
Morymidae							
*Hyperopisus bebe* sp1	*Brevimyrus niger*	2	C/A				
*H.bebe* sp2	*B. niger*	2	C/A				
*Marcusenius senegalensis*	*Marcusenius senegalensis*	5	BOLD: AAL6601	0.68	1.23	3.21	*Marcusenius macrolepidotus*
*Mormyrops anguilloides*	*M. anguilloides*	3	BOLD: ACR9609	0.34	0.34	4.33	*Mormyrops masuianus*
*Mormyrus rume*	*Mormyrus hasselquistii*	1	C/A				
*Mormyrus macrophthalmus*	*Mormyrus macrophthalmus*	2	BOLD: AAM0703			8.83	*Mormyrus caballus*
*Mormyrus rume*	*Mormyrus tapirus*	2	C/A				
Protopteridae							
*Protopterus* sp.	*Protopterus* sp.	1	BOLD: AAL6244	0.51	0.77	2.56	*Protopterus* sp.
Schilbeidae							
*Schilbe intermedius*	*S. intermedius*	9	BOLD: AAL5704	0.44	0.77	6.1	*S. intermedius*
*Schilbe* sp.	*S. intermedius*	1	BOLD: AAD0083	0.17	0.17	5.08	*S. intermedius*
*Schilbe mystus*	*Schilbe mystus*	2	BOLD: AAM0039	N/A	N/A	4.82	*Schilbe grenfelli*
Tetraodontidae							
*Tetraodon lineatus*	*Tetraodon lineatus*	3	C/A				

*Note*

*AvD*: average intraspecific distance; BIN: Barcode Index Number, an identification number for barcoding clusters recognized by BOLD within the species; C/A: individuals for which a species level match could not be made; *DNN*: average genetic distance to the nearest neighbor; *MxD*: maximum intraspecific distance; *N*: number of barcode sequences; N/A: sequences for the specimen are not available in BOLD.

### 3.4 Mismatch in taxonomy

Of the 53 morphologically identified species, 34 (64.20%) matched with species names assigned using morphological approach and GenBank database. Using BOLD database, only 19 (35.80%) were in accordance with species names assigned using both morphological and BOLD database (Table 2). We observed cases of mismatch in names assigned to species using morphology and DNA barcoding approach. While species level assignments of 21 species (39.60%) were in accordance with species level identification made using morphology and DNA barcoding approach, our result revealed mismatch in species names assigned to 27 species (50.90%) using morphology and DNA barcoding. Comparing GenBank and BOLD databases, we encountered four (7.50%) cases of mismatches in which our query sequence for GenBank and BOLD showed a sequence match with different species names within 97% similarity cutoff (Table 2).

### 3.5 Tree‐based identification

**Figure 1:**
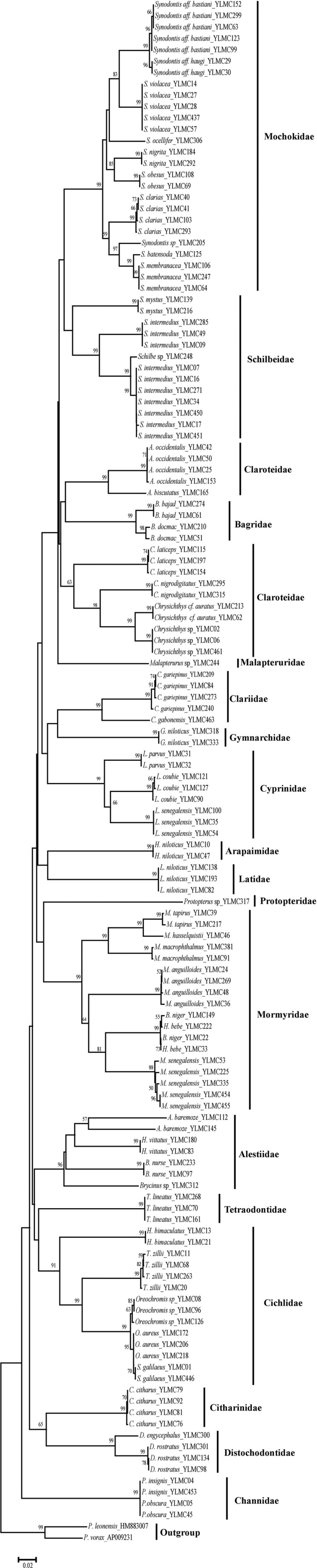
Neighbor‐Joining tree of Cytochrome *c* oxidase I gene sequences derived from 53 freshwater fish species from north‐central Nigeria. Values above branches are bootstrap values >50% and values below 50% are not shown

We used HM883007 (*Pellonula leonensis*); and AP009231 (*Pellonula vorax*) as the out‐group taxa to root the NJ tree (NJ) for the pooled *COI* sequences of freshwater fishes from north‐central Nigeria (Figure 1). From our NJ tree analyses, most individuals of same species (92.50%) clustered together. Most of the species clusters in the NJ tree were strongly supported (bootstrap values ≥95%) except for *Alestes baremoze* (bootstrap values = 57%). Furthermore, undescribed species (*Chrysichythys* sp., *Malapterurus* sp., *Protopterus* sp., *Synodotis* sp., and *Schilbe* sp.) formed strongly supported lineages in the NJ tree and were clearly separated from their sister species (Figure 1). Thus, the NJ tree revealed that species identification based on morphological evidence and molecular methods are broadly consistent in most cases.

The K2P intergeneric *COI* sequence divergence values ranged from 0.30% to 31.40% (Table 4). The smallest intergenera genetic divergence values were observed between *Hyperopisus* and *Brienomyrus* (0.30%); while the highest pairwise comparison was between *Protopterus* and *Lates* (31.40%) and between *Parachanna* and *Malapterurus* (31.30%). We obtained interspecific divergence in the range of 0.30%–32.40% (Table S2). The least divergence (0.30%) was observed between *Hyperopisus bebe* and *Brienomyrus niger;* while highest interspecific divergence (32.40%) was between *Bagrus bajad* and *Protopterus* sp. (Table S2). Intraspecific genetic distances range from 0.00% and 16.39% (Table S3). We observed the highest intraspecific divergence in *A. baremoze* (16.39%) and *Schilbe intermedius* (4.14%) (Table S3).

**Table 4:** Intergeneric pairwise genetic distance (%) of *COI* sequence data of freshwater fishes from north‐central Nigeria using Kimura‐2‐parameter

S/NO	Genus	1	2	3	4	5	6	7	8	9	10	11	12	13	14	15	16	17	18	19	20	21	22	23	24	25	26	27
1	*Alestes*																											
2	*Auchenoglanis*	28.40																										
3	*Bagrus*	26.70	18.60																									
4	*Brienomyrus*	27.80	28.10	28.70																								
5	*Brycinus*	17.90	23.70	24.60	24.40																							
6	*Chrysichthys*	25.90	19.80	18.90	28.40	24.20																						
7	*Citharinus*	24.70	23.00	26.50	28.00	22.70	23.70																					
8	*Clarias*	25.60	22.90	19.90	24.30	24.30	22.40	26.30																				
9	*Clarotes*	24.60	21.30	20.00	27.90	24.10	17.60	23.50	21.00																			
10	*Distichodius*	27.30	27.80	24.70	27.90	24.50	27.80	20.60	23.50	26.00																		
11	*Gymnarchus*	28.10	27.00	25.60	26.30	27.90	27.90	30.20	22.40	26.30	26.90																	
12	*Hemichromis*	26.40	25.60	26.80	28.00	25.30	24.20	24.60	26.90	24.20	27.30	28.40																
13	*Heterotis*	28.70	27.00	26.30	25.50	27.80	30.10	27.00	27.90	25.90	26.20	24.80	24.30															
14	*Hydrocynus*	17.20	25.00	25.60	26.10	19.10	25.00	24.80	22.20	26.20	26.00	27.10	25.10	27.50														
15	*Hyperopisus*	27.60	27.80	28.40	0.30	24.20	28.40	27.90	24.20	27.60	27.60	26.30	27.70	25.30	25.90													
16	*Labeo*	28.50	27.40	25.20	24.60	26.70	25.30	25.00	23.30	23.70	24.20	24.10	26.40	25.20	27.70	24.60												
17	*Lates*	28.20	27.90	27.60	25.30	26.10	27.90	27.20	27.80	28.70	24.20	27.40	28.60	24.60	27.20	25.00	27.90											
18	*Malapterurus*	26.90	20.30	21.40	25.70	23.80	22.80	24.00	21.60	19.70	22.60	27.30	28.70	24.00	28.00	25.60	24.90	27.00										
19	*Marcusenius*	27.80	26.70	27.50	12.70	24.50	28.10	28.20	23.10	26.40	26.10	25.50	27.70	27.50	26.00	12.70	25.30	25.50	24.40									
20	*Mormyrops*	30.20	26.90	29.60	17.30	27.60	27.70	27.60	28.00	28.30	29.80	26.40	27.40	27.70	28.10	17.30	27.00	29.90	27.00	18.50								
21	*Oreochromis*	25.40	24.70	25.10	27.60	26.10	21.60	22.70	25.40	23.30	25.90	27.80	18.30	24.60	23.40	27.30	24.60	25.90	27.50	27.70	25.20							
22	*Parachanna*	30.00	25.90	28.70	29.70	25.80	29.10	25.10	31.20	28.10	27.40	28.90	25.90	28.50	27.00	29.60	26.90	27.70	31.30	30.50	28.80	24.80						
23	*Protopterus*	31.00	28.60	31.00	29.20	29.10	29.80	29.60	29.30	28.20	30.10	28.90	29.30	30.30	27.30	28.90	31.40	28.60	29.80	30.10	28.60	26.70	33.10					
24	*Sarotherodon*	25.70	24.70	24.50	28.20	26.50	21.80	22.70	25.60	23.80	25.30	28.40	18.60	24.30	23.70	27.90	24.60	26.20	27.40	28.30	25.50	0.50	24.90	27.00				
25	*Schilbe*	24.70	17.30	19.40	26.40	23.00	19.90	24.40	21.40	21.70	25.50	25.60	25.00	24.60	22.90	26.20	24.10	23.20	20.50	25.00	25.30	22.50	27.10	29.20	22.70			
26	*Synodontis*	26.70	19.30	22.10	26.70	24.30	20.30	23.20	22.40	20.50	26.40	26.20	24.80	24.70	23.40	26.70	24.50	25.30	19.70	26.50	25.50	23.70	28.70	29.90	24.00	16.80		
27	*Tetraodon*	27.70	24.90	25.70	27.10	24.50	26.00	26.60	27.80	27.10	27.00	27.10	24.40	26.90	22.50	26.80	24.50	27.10	25.80	27.30	25.50	23.60	26.50	30.80	23.90	26.60	25.60	
28	*Tilapia*	26.00	26.20	26.20	25.70	25.10	22.80	21.70	26.80	24.10	28.90	28.30	19.00	25.30	21.70	25.70	24.40	26.80	28.50	24.40	26.10	12.90	24.80	29.50	13.10	25.40	24.70	22.90

### 3.6 Applications of DNA barcode reference data

#### 3.6.1 Identification of unknown fish tissue samples

**Figure 2:**
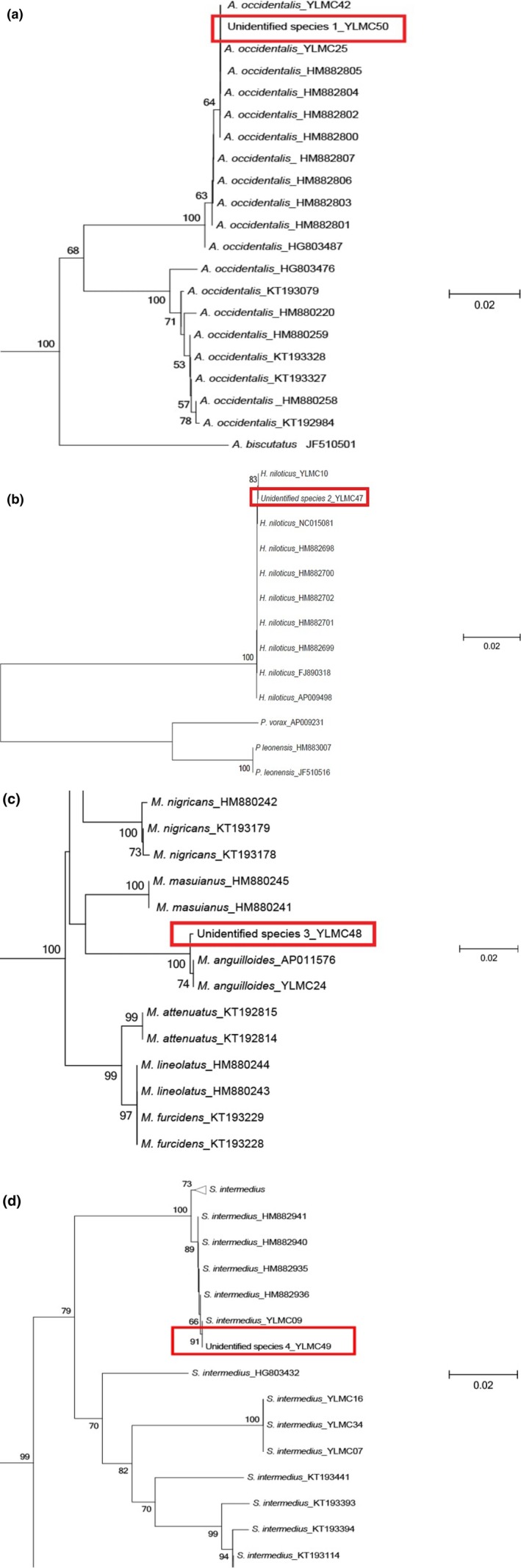
Parts of the neighbor‐joining tree of Cytochrome *c* oxidase I (*COI*) used to identify (a) unknown tissue 1 (b) unknown tissue 2 (c) unknown tissue 3 (d) unknown tissue 4. Values above branches are bootstrap values >50% and values below 50% are not shown

All sequences of the four unknown fish samples collected from fishermen were successfully amplified. Our query search of *COI* sequences of unidentified species 1 and 2 in GenBank showed 100% sequence similarity with *Auchenoglanis occidentalis* (HM882800) and *Heterotis niloticus* (FJ890318), respectively. The NJ tree analysis clustered unidentified species 1 with *A. occidentalis* from Rivers Moro and Niger of north‐central Nigeria (Figure 2a). Furthermore, unidentified species 2 clustered with *H. niloticus* from River Asa (Fig. 2b). Unidentified species 3 and 4 showed, respectively, 99% and 100% DNA sequence similarity with *Mormyrops anguilloides* (AP011576) and *S. intermedius* (HM882935). In addition, unidentified species 3 and 4 clustered with *M. anguilloides* from Rivers Moro and Niger (Fig. 2c), and *S. intermedius* from River Asa (Fig. 2d), respectively. Therefore, DNA barcoding could aid in identification of unknown tissue samples. These unknown tissues samples could possibly be from fish collected from rivers across Nigeria.

#### 3.6.2 Uncovering cryptic diversity

**Figure 3:**
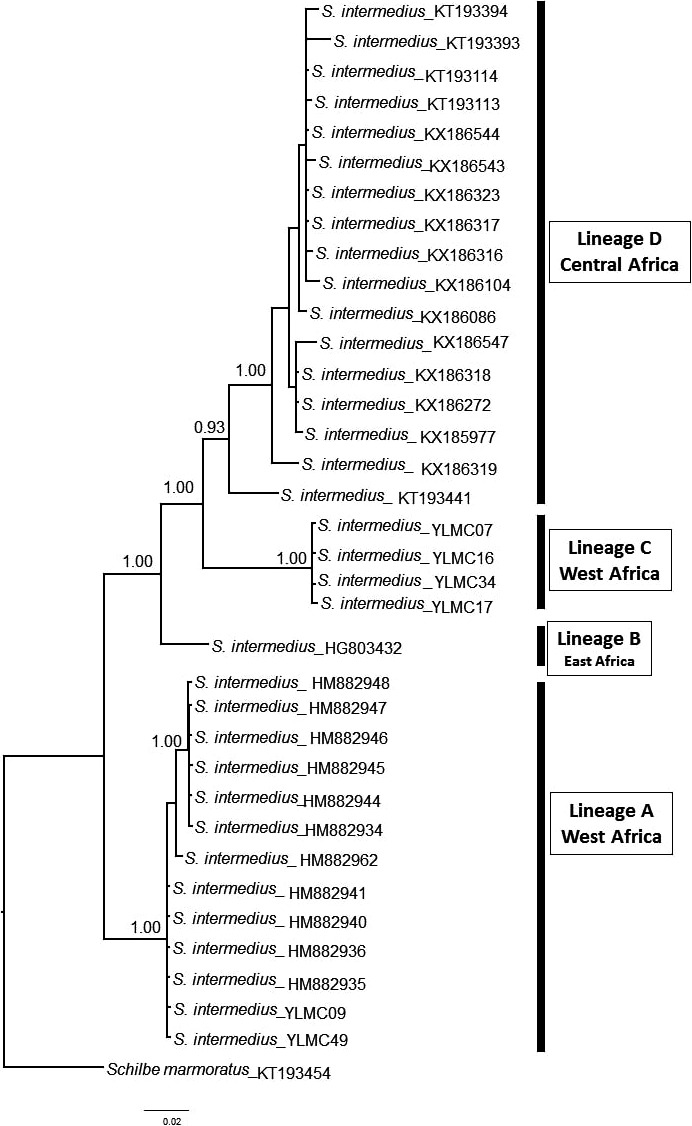
Matrilineal genealogy of *Schilbe intermedius* based on the Bayesian inference of *COI* sequences. Values above branches are Bayesian posterior probabilities (PP ≥ 0.95) and values below (PP < 0.95) are not shown

Our NJ tree‐based analyses of *COI* sequences of freshwater fishes from north‐central Nigeria revealed that populations of *S. intermedius* consist of at least two distinct lineages (bootstrap values = 99%, Fig. 1). This illustrates possibility of cryptic lineage diversity within Nigerian *S. intermedius*. To test for this, we downloaded 29 sequences of *S. intermedius* from the GenBank (Table S1). The BI analysis was rooted with *Schilbe marmoratus* (GenBank no. KT193454) as out‐group taxon Our BI analysis recovered four lineages (A–D) within *S. intermedius* (Figure 3), with moderate to high support. Lineages corresponded greatly to geography. Lineage A consists of samples distributed in West Africa (north‐central and southeastern Nigeria); lineage B includes samples from East Africa (Mozambique); lineage C restricted to individuals from north‐central Nigeria; while lineage D includes individuals widely distributed in Central Africa (Congo). Based on this result, these lineages were treated as discrete units and the pairwise level of divergence was recalculated. The results showed that the levels of divergence among the lineages were higher than 3% (Table 5). Highest pairwise level of divergence (9.348%) was between the two West African (Nigerian) lineages (lineage A and C) and the least pairwise divergence (6.698%) was between East and Central Africa (Table 5). Intraspecific genetic distances in all groups (except lineage B represented by one individual) fell between 0.091% and 1.31% (Table 5).

**Table 5:** Estimates of mtDNA (*COI*) evolutionary divergence (%) over sequence pairs between lineages of *Schilbe intermedius* using Kimura‐2‐parameter

	Lineage A	Lineage D	Lineage C
Lineage A			
Lineage D	8.419		
Lineage C	9.348	7.439	
Lineage B	6.868	6.698	7.049

## 4 DISCUSSION

In our study, DNA barcoding approach was very efficient in species identification. The success rates of DNA barcoding approach in our study (95.60%) was higher than the 93% success rate reported for Canadian freshwater fish (Hubert et al., 2008) and the 90% success rate reported for North American freshwater fish (April, Mayden, Hanner, & Bernatchez, 2011). However, our DNA barcoding success rate was lower when compared to 100% success rate reported by Shen, Guan, Wang, and Gan (2016) and 98.30% success rate reported for Indian freshwater fishes (Lakra et al., 2015). In most cases, our study shows that *COI* sequences effectively clustered most of the conspecific and congeneric species. This was also observed in similar studies in fishes from Upper Parana River Basin (Pereira, Hanner, Foresti, & Oliveira, 2013), freshwater fishes from southeastern Nigeria (Nwakanma et al., 2015; Nwani et al., 2011) and freshwater fishes from southwestern Nigeria (Falade, Opene, & Benson, 2016). In the studies of Nwani et al. (2011), 70 species of the southeastern Nigeria were well identified using DNA barcoding approach. Most of the species recorded in our present study have been studied using DNA barcoding approach by Falade et al. (2016), Nwakanna et al. (2015), and Nwani et al. (2011). These previous studies represent a potential reference database for the identification of Nigerian ichthyofaunal diversity. This may have accounted for the high identification success observed in our study.

Furthermore, we verified the taxonomic reliability of DNA barcoding against traditional method. Although the traditional method was effective in assigning species names to individuals, yet, this method is always tedious and time consuming, and often requires collaboration of taxonomists to share their expertise and confirm the identity of these species. We observed cases where species names assigned using morphological methods did not agree with names assigned using DNA barcoding approach. Such misidentification is a common challenge in taxonomy especially for morphologically similar species and accurate identification of the species also relies on the level of expertise of the taxonomist. Our study therefore gives strong evidence of integrating morphological and molecular methods in ichthyofaunal studies. However, because Nigerian ichthyofauna are facing threats arising from both climatic change and pollution of water bodies, the use of genetic methods, for example, DNA barcoding, may facilitate species identification. Most Nigerian ichthyofauna are still under‐studied and identification keys are often lacking. Thus, the combined use of morphological and genetic (DNA barcoding) data will aid in the identification of fish species in this region. Hence, developing a complete DNA barcode reference library for Nigerian ichthyofauna will facilitate taxonomy and biodiversity research in this region.

### 4.1 Application of DNA barcoding reference data

We reported two applications of DNA barcoding: identifying unknown samples from fishermen and uncovering cryptic diversity. In the case of the identification of unknown samples, DNA barcode reference data were very useful in identifying the unknown fish samples. Hence, the acquisition of DNA barcoding data will aid in species identification, which in turn, help in the conservation and management planning of Nigerian fishery resources. In addition, DNA barcoding approach could be a relevant tool for identifying unknown samples for wildlife‐related law enforcement and resolving a civil suit (Jeong, Byeung, Ki, & Su, 2013). It is obvious that the regulatory use of DNA barcoding, as suggested in this study, would be effective if most of the Nigerian freshwater fishes are documented in sequence libraries. This therefore calls for DNA barcoding of more species from other regions in Nigeria that would assist in identification and management of Nigerian freshwater fishes.

Although the primary goal of DNA barcoding is to identify species, intraspecific phylogeographic structure became evident in our study. This reveals applicability of DNA barcoding in uncovering cryptic diversity within species. Detecting cryptic species from molecular biodiversity inventories for many systematic biologists is the most appealing application of DNA barcoding (Knebelsberger et al., 2014). Populations of *S. intermedius* collected during our field survey were morphologically similar and their identification was controversial. However, in our study, DNA barcoding discriminated *S. intermedius* population from north‐central Nigeria into two distinct clusters with intraspecific divergence of 4.18%. Our finding was consistent with previous studies (e.g., Benzaquem et al., 2015; Mat Jaafar et al., 2012; Mohammed et al., 2016; Van der Bank, Greenfield, Daru, & Yessoufou, 2012) that showed the effectiveness of DNA barcoding in uncovering cryptic lineage diversities in fishes. There is the possibility that some of the identified lineages exhibit minute morphological differences that may have been overlooked in the past. However, due to the high rate of biodiversity loss, the distinct lineages uncovered from our study require consideration for conservation strategies and fishery management practice (Fraser & Bernatchez, 2001).

Comparison of our *COI* sequences with others from GenBank revealed existence of several more complexes of potentially cryptic lineages within *S. intermedius*. Contrary to previous studies (Nwani et al., 2011) that hypothesized two lineages of *S. intermedius* in Nigeria, our study revealed the presence of more than two lineages within this species in Nigeria. Increasing sample size and geographic sampling range may uncover more cryptic diversity within *S. intermedius*. Thus, our data is insufficient to explore the hypothesis of speciation within *S. intermedius*. To explore this hypothesis, it is necessary to sample these species across broad geographic range. Careful examination of possible morphological variations and more genetic analyses would aid in determining whether the detected cryptic lineages be warranted species status. Thus, our study emphasizes the need for a more complete reference DNA barcode data across Nigeria for the detection of more cryptic diversity in freshwater fish.

### 4.2 Reliability of DNA barcode reference data

The success of using DNA barcoding approach for species identification relies on the availability of high‐quality reference sequences in public sequence libraries such as GenBank and BOLD. Several sequences from the databases, particularly for individuals under the genera *Oreochromis*,* Parachanna*,* Hyperopisus* and *Brienomyrus* may require further taxonomic validation. In line with the findings of Becker, Hanner, and Steinke (2011), possible taxonomic errors exist for sequences submitted to databases. Possible sources of these errors might be due to either morphological misidentifications of the voucher specimen, contamination during sample processing in the laboratory, insufficient taxonomic identification or synonym and syntax problems (Radulovici, Archambault, & Dufresne, 2010; Tautz, Arctander, Minelli, Thomas, & Vogler, 2003; Ward, 2012). Accurate taxonomic review of already published DNA barcode data would be relevant in resolving such issues. This will increase the reliability of international barcode reference libraries like GenBank and BOLD.

## 5 CONCLUSION

Our study demonstrates the usefulness of DNA barcoding for the identification of fish species in north‐central Nigeria and uncovering lineage diversity. This study contributes to the construction of DNA reference barcode data for Nigerian fish fauna. This study has therefore contributed important data for the species identification, which in turn will aid the management of freshwater fishes in Nigerian inland water bodies. Furthermore, it has provided additional data to the major databases of GenBank and BOLD. We also confirm that DNA barcoding could assist in resolving issue of ambiguousness in identification of morphologically similar species. Thus, this approach could assist in the discovery and characterization of closely related species. However, we recommend further validation of GenBank sequences with respect to their voucher specimen to prevent future misidentification of fish species. In addition, this study underscores the relevance of combined use of morphological and genetic (DNA barcoding) data in the identification of fish species. Furthermore, our results demonstrate the application of DNA barcode reference data in uncovering cryptic diversity within *S. intermedius*. Finally, we recommend DNA barcode approach in species identification, ichthyofaunal studies, conservation and management planning of Nigerian fishery resources.

## CONFLICT OF INTEREST

None declared.

## AUTHORS’ CONTRIBUTIONS

O.A.I., L.M.N., M.K.M., S.O.O., and A.C.A. designed the study; S.O.O. collected and preserved the samples; L.M.N., A.C.A., Y.Y.W., J.C., and W.Z.W. performed the molecular laboratory work and generated the sequence data; L.M.N performed the genetic and morphological analyses; A.C.A provided technical assistance for the study; L.M.N., O.A.I., and S.O.O. wrote the initial draft of the manuscript; M.K.M., C.G.N., I.C.N., A.O.A., C.D.N., O.A.U., A.A.A.U., E.O.F., and A.C.A. critically revised the manuscript. All authors read and approved the final manuscript.

## DATA ACCESSIBILITY

DNA sequences: GenBank Accession Nos MG824552–MG824685; for each individual, details on locality information and GenBank Accession no. for its sequence data are shown in Table 1.
