# Supplementary material for: DNA barcoding of economically important freshwater fish species from north‐central Nigeria uncovers cryptic diversity
Source: Ecol Evol. 2018 Jun 13;8(14):6932–51. doi: 10.1002/ece3.4210 (PMC6065348; doi:10.1002/ece3.4210)
Supplement: Supplementary file 1 [file ECE3-8-6932-s001.docx]

Table S1: Sequences of retrieved from the GenBank for the phylogenetic reconstruction of *Schilbe intermedius.*

| Species name | Genbank Accession Number | Locality |
| --- | --- | --- |
| Ingroup |  |  |
| *S. intermedius* 1 | HM882934 | West Africa, Nigeria, South-East |
| *S. intermedius* 2 | HM882935 | West Africa, Nigeria, South-East |
| *S. intermedius* 3 | HM882936 | West Africa, Nigeria, South-East |
| *S. intermedius* 4 | HM882940 | West Africa, Nigeria, South-East |
| *S. intermedius* 5 | HM882941 | West Africa, Nigeria, South-East |
| *S. intermedius* 6 | HM882944 | West Africa, Nigeria, South-East |
| *S. intermedius* 7 | HM882945 | West Africa, Nigeria, South-East |
| *S. intermedius* 8 | HM882946 | West Africa, Nigeria, South-East |
| *S. intermedius* 9 | HM882947 | West Africa, Nigeria, South-East |
| *S. intermedius* 10 | HM882948 | West Africa, Nigeria, South-East |
| *S. intermedius* 11 | HM882962 | West Africa, Nigeria, South-East |
| *S. intermedius* 12 | HG803432 | East Africa, Mozambique |
| *S. intermedius* 13 | KT193441 | Central Africa, Democratic Republic of Congo |
| *S. intermedius* 14 | KT193393 | Central Africa, Democratic Republic of Congo |
| *S. intermedius* 15 | KT193394 | Central Africa, Democratic Republic of Congo |
| *S. intermedius* 16 | KX193113 | Central Africa, Democratic Republic of Congo |
| *S. intermedius* 17 | KX193114 | Central Africa, Democratic Republic of Congo |
| *S. intermedius* 18 | KX186543 | Central Africa, Democratic Republic of Congo |
| *S. intermedius* 19 | KX186544 | Central Africa, Democratic Republic of Congo |
| *S. intermedius* 20 | KX186323 | Central Africa, Democratic Republic of Congo |
| *S. intermedius* 21 | KX186316 | Central Africa, Democratic Republic of Congo |
| *S. intermedius* 22 | KX186317 | Central Africa, Democratic Republic of Congo |
| *S. intermedius* 23 | KX186104 | Central Africa, Democratic Republic of Congo |
| *S. intermedius* 24 | KX186086 | Central Africa, Democratic Republic of Congo |
| *S. intermedius* 25 | KX186547 | Central Africa, Democratic Republic of Congo |
| *S. intermedius* 26 | KX186318 | Central Africa, Democratic Republic of Congo |
| *S. intermedius* 27 | KX186319 | Central Africa, Democratic Republic of Congo |
| *S. intermedius* 28 | KX186272 | Central Africa, Democratic Republic of Congo |
| *S. intermedius* 29 | KX185977 | Central Africa, Democratic Republic of Congo |
|  |  |  |
| Outgroup |  |  |
| *Schilbe marmoratus* | KT193454 | Loboya River, Democratic Republic of Congo |
